# Latent Profiles of Informal Social Contact and Their Associations with Physical Exercise Participation Among Young Adults in China

**DOI:** 10.3390/bs16060883

**Published:** 2026-06-01

**Authors:** Tianci Lu, Hanwen Chen, Baole Tao, Jun Yan

**Affiliations:** College of Physical Education, Yangzhou University, Yangzhou 225127, China; dx120240094@stu.yzu.edu.cn (T.L.); dx120230091@stu.yzu.edu.cn (H.C.); dx120190064@yzu.edu.cn (B.T.)

**Keywords:** Chinese General Social Survey (CGSS), latent profile analysis, young adults, informal social contact, physical exercise participation frequency

## Abstract

Physical inactivity remains a major public health concern worldwide, including among young adults. Drawing on data from the 2021 China General Social Survey (CGSS), this study examined heterogeneous patterns of informal social contact and their associations with exercise frequency among an analytic sample of young adults aged 18–35 years in China. A total of 1853 respondents were included in the analysis. Latent profile analysis identified four distinct informal social contact profiles: a generally low-contact profile, a relative-oriented and low-neighbor contact profile, a neighbor-oriented profile with approximately average friend contact, and a broadly high-contact profile. Multinomial logistic regression was used to examine associations between exercise frequency, sociodemographic characteristics, and profile membership. The BCH method with Holm–Bonferroni correction was used to compare exercise frequency across profiles. The results showed that rural respondents were more likely to belong to the broadly high-contact profile, whereas respondents who never exercised were more likely to belong to the generally low-contact profile. After correction for multiple BCH comparisons, only the difference between the relative-oriented, low-neighbor contact profile and the neighbor-oriented, average-friend contact profile remained statistically significant, with the former reporting higher exercise frequency. These findings highlight heterogeneity in informal social contact within the analytic sample and suggest that different configurations of everyday contact with relatives, friends, and neighbors are associated with exercise frequency.

## 1. Introduction

Social support refers to the resources, assistance, and interpersonal connections that individuals obtain through their social relationships and networks (Sarason et al., 1983; Uehara, 1990). It is commonly distinguished between formal support, which is provided through institutions, public programs, or social security systems, and informal support, which is embedded in everyday relationships with family members, friends, neighbors, and peers. Research has shown that informal social support, such as family and peer support, plays a significant role in improving physical health by influencing individuals’ physical exercise behaviors (Sarkar et al., 2016; Scarapicchia et al., 2017; Cao et al., 2023). This support can motivate individuals to adopt regular physical activity, particularly when social ties encourage or facilitate participation. In recent years, this perspective has gained increasing policy relevance in China. National strategies such as the National Fitness Program (2021–2025) (The State Council of the People’s Republic of China, 2021) and the Healthy China 2030 (The State Council of the People’s Republic of China, 2016) initiative emphasize strengthening grassroots exercise infrastructure and social support networks to promote physical activity among the general population, especially young people. However, formal policies and public exercise programs do not necessarily translate into regular exercise participation in everyday life. For young adults, exercise behavior may be shaped not only by access to public facilities, but also by time constraints, peer interaction, residential context, and daily social routines. Informal relationships with relatives, friends, and neighbors may therefore provide everyday contexts in which exercise opportunities, companionship, and social participation occur. Factors such as geographic location and local policy disparities, especially in rural areas, can limit access to these formal structures, further highlighting the importance of informal social networks. For example, individuals in urban areas have easier access to physical exercise facilities and opportunities than those in rural areas (Moreno-Llamas et al., 2023), and poorly developed rural sports facilities may also discourage individuals from participating in physical exercise (Zheng & An, 2015). It is important to note, however, that the CGSS items used in the present study capture the frequency of contact and social recreational activities with relatives, friends, and neighbors, rather than direct emotional, instrumental, informational, or appraisal support. Therefore, in the empirical analysis, these indicators are referred to as informal social contact rather than informal social support. Accordingly, this study focuses on the relationship between informal social contact and exercise frequency among young adults. Referring to the research design of Yang et al. (2023), informal social contact is operationalized as contact with relatives, contact with friends, and contact with neighbors.

Understanding exercise participation requires consideration of motivational mechanisms and the social opportunities that enable regular behavior. Michie proposed the ‘Capability Opportunity Motivation-Behavior’ theory, known as the COM-B theory (Michie et al., 2011), which suggests that sustainable behavioral change results from the interaction of capability, opportunity, and motivation. Building on this, Meng Xiaoping developed a framework relating sport ability, participation opportunity, motivation, and behavior (Meng & Zhang, 2022). These elements influence whether individuals engage in sport and how frequently they do so. For adults, sport motivation has been shown to have the most substantial effect on sport behavior, including participation frequency, compared to ability and opportunity (Howlett et al., 2019). In the context of exercise frequency, informal social contact may be understood as part of the opportunity structure surrounding physical activity. It may create opportunities for companionship, encouragement, shared routines, and participation in social or recreational activities, all of which may be associated with more regular exercise participation.

A growing body of research suggests that social support is positively associated with physical activity and exercise participation (Sarkar et al., 2016; Ji et al., 2022; Hailey et al., 2023). However, much of this evidence has examined average associations between social support and physical activity, which may obscure heterogeneity in how different sources of everyday contact cluster within individuals. Latent profile analysis provides a person-centered approach for identifying subgroups of individuals with similar response patterns across multiple indicators (Yin et al., 2020). Such person-centered methods are useful when the goal is to examine heterogeneity in psychosocial or behavioral characteristics rather than to estimate only variable-level associations. Previous exercise-related research has also used latent profile approaches to identify heterogeneous motivational or behavioral patterns among participants (Emm-Collison et al., 2020). Therefore, this study uses latent profile analysis to identify informal social contact profiles among young adults and to examine how these profiles are associated with exercise frequency. By focusing on configurations of contact with relatives, friends, and neighbors, this study aims to provide a more nuanced understanding of the everyday social contexts associated with exercise participation among young adults.

## 2. Materials and Methods

### 2.1. Ethics Approval

This study used publicly available, anonymized data from the CGSS 2021. Because the dataset contains no identifiable personal information, additional institutional ethical approval was not required.

### 2.2. Data Sources

The data for this study are drawn from the public database of the 2021 China General Social Survey (CGSS), a large-scale national survey jointly conducted by the National Survey Research Center at Renmin University of China and other academic institutions. Initiated in 2003, the CGSS is China’s earliest nationwide, continuous, and comprehensive academic survey project, aiming to monitor changes in Chinese society and living conditions. The 2021 CGSS employed a multi-stage sampling design, covering 19 provinces, autonomous regions, and municipalities across China. The sample was drawn to reflect the demographic and geographic distribution of the national population, and included both urban and rural areas. Data were collected through face-to-face interviews using standardized questionnaires administered by trained fieldworkers.

### 2.3. Research Subjects

**Figure 1:**
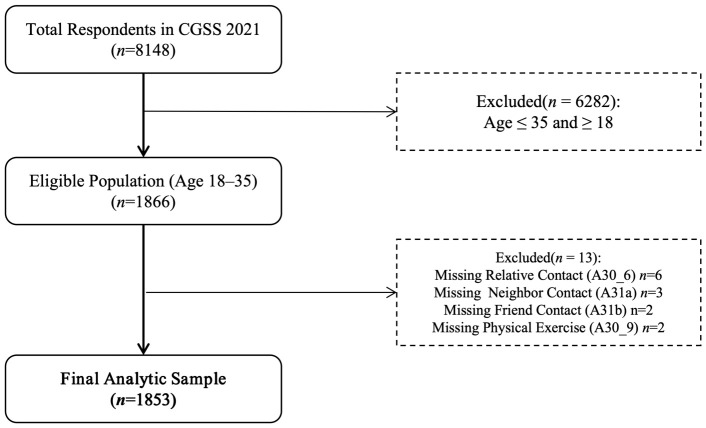
Sample selection flowchart.

The study focused on young adults in China. According to the Medium- and Long-Term Youth Development Plan (2016–2025) issued by the State Council (2017), young people are broadly defined as those aged 14–35 years. However, because the CGSS 2021 surveyed adults only, the present study defined young adults as respondents aged 18–35 years at the time of the survey. Accordingly, respondents born between 1986 and 2003 were considered eligible for inclusion. The CGSS 2021 included 8148 respondents in total. Of these, 1866 respondents were aged 18–35 years and were therefore eligible for this study. Among the age-eligible respondents, 13 cases were excluded due to missing data, including missing values on relative contact (*n* = 6), neighbor contact (*n* = 3), friend contact (*n* = 2), and exercise frequency (*n* = 2). Missing data were handled using complete-case analysis (listwise deletion). Because only 13 age-eligible respondents were excluded due to missing values, accounting for 0.70% of the age-eligible sample, formal comparisons between included and excluded cases were not conducted due to the limited interpretability of statistical tests based on such a small excluded group. The final analytic sample retained 99.30% of all age-eligible respondents. The final analytic sample therefore consisted of 1853 young adults. Sampling weights were not applied in the primary analyses. Therefore, the estimated profile proportions and associations should be interpreted with caution and understood as sample-specific rather than nationally representative. The sample selection process is presented in Figure 1.

### 2.4. Variable Measurement and Data Processing

This study examined the associations between informal social contact profiles and exercise frequency among young adults. Gender, age, household registration status, educational attainment, income, and work characteristics were included as covariates because these factors may be associated with both social contact patterns and exercise participation. The survey items used in this study were taken from the CGSS 2021 questionnaire. Variable measurements and coding procedures are summarized in Table 1.

The main indicators for latent profile analysis were three dimensions of informal social contact: contact with relatives, contact with neighbors, and contact with friends. These indicators were derived from four CGSS 2021 items:

(1) In the past year, did you often get together in your free time with relatives who did not live with you?
(2) How often do you engage in social recreational activities with your neighbors?
(3) How often do you engage in social recreational activities with other friends?
(4) In the past year, did you often meet with friends in your free time?

The first item was used to measure contact with relatives, the second item was used to measure contact with neighbors, and the third and fourth items were used to construct the indicator of contact with friends. These items reflect the frequency of contact or social recreational activities rather than the functional content of social support. Therefore, they were treated as indicators of informal social contact. Items were reverse-coded when necessary so that higher scores indicated more frequent contact. Because the response scales differed across items, response categories were harmonized before analysis. Specifically, the categories “about once a month” and “a few times a month” in the friend social-recreational activity item were combined, and “a few times a year” and “once a year or less” in the friend-meeting item were combined. The informal social contact indicators were then standardized before latent profile analysis to reduce the influence of scale differences across indicators.

Exercise frequency was measured using the item “In the past year, how often did you participate in physical exercise during your free time?” This item was reverse-coded so that higher scores indicated more frequent exercise participation. It was used as a predictor in the multinomial logistic regression and as the distal outcome in the BCH analysis.

Sociodemographic covariates were processed as follows. Gender was coded as male = 1 and female = 0. Household registration status was coded as rural = 1 and non-rural/resident registration = 0; military status, other status, and no household registration were treated as missing because of very small cell sizes. Educational attainment was recoded into seven ordered categories, ranging from no formal education to postgraduate education or above. Detailed recoding rules for educational attainment are presented in Table 1. For income, special CGSS codes indicating inapplicable responses, “don’t know,” refusal, or extreme non-substantive values were treated as missing, and valid annual income was transformed using the natural logarithm. Work characteristics were measured using two items on physically demanding work and mentally demanding work, which were reverse-coded for consistency. All variable coding procedures are reported in Table 1.

### 2.5. Data Analysis

The data analysis proceeded in three steps. First, latent profile analysis was used to identify patterns of informal social contact among young adults. Second, multinomial logistic regression was conducted to examine whether profile membership was associated with exercise frequency and sociodemographic covariates. Third, the BCH method was used to compare exercise frequency across the identified profiles while accounting for classification uncertainty.

Latent profile analysis was conducted in Mplus 8.3 using the standardized indicators of contact with relatives, contact with neighbors, and contact with friends. Models with two to five profiles were estimated sequentially to determine the optimal number of profiles. Following previous studies (Nylund et al., 2007; Emm-Collison et al., 2020), model selection was based on multiple fit indices, including the Akaike information criterion (AIC), Bayesian information criterion (BIC), sample-size adjusted Bayesian information criterion (ABIC), Lo–Mendell–Rubin likelihood ratio test (LMR), bootstrap likelihood ratio test (BLRT), and entropy. Lower AIC, BIC, and ABIC values indicate better relative model fit, whereas higher entropy values indicate clearer classification. Significant LMR and BLRT results suggest that a model with k profiles fits the data better than a model with k − 1 profiles. In addition to these statistical indices, profile size, classification quality, and substantive interpretability were also considered when selecting the final model.

After the optimal latent profile solution was selected, multinomial logistic regression was used to examine factors associated with profile membership. In this model, latent profile membership was treated as the dependent variable, whereas exercise frequency and sociodemographic variables, including gender, age, household registration status, educational attainment, income, and work characteristics, were entered as predictors. The broadly high-contact profile was used as the reference category.

Finally, differences in exercise frequency across latent profiles were examined using the BCH method in Mplus 8.3. The BCH method allows distal outcome comparisons across latent profiles while retaining individuals’ classification uncertainty. Pairwise comparisons of exercise frequency across profiles were adjusted using the Holm–Bonferroni correction to reduce the risk of inflated Type I error due to multiple comparisons.

## 3. Results

### 3.1. Results of Latent Profile Analysis of Informal Social Contact Among Young Adults

The results of the latent profile analysis are presented in Table 2. Models with two to five profiles were estimated and compared using multiple fit indices, including AIC, BIC, sample-size adjusted BIC, entropy, LMR, and BLRT. The AIC, BIC, and sample-size-adjusted BIC values decreased as the number of profiles increased and reached their lowest values in the five-profile model. However, the LMR for the five-profile model was not statistically significant, indicating that adding a fifth profile did not provide a significant improvement over the four-profile model.

Additional diagnostic results also supported the retention of the four-profile solution. The four-profile model showed higher entropy than the five-profile model (0.917 vs. 0.834), suggesting better classification accuracy. Moreover, the smallest class in the four-profile solution accounted for 11.93% of the sample, whereas the smallest class in the five-profile solution accounted for only 6.04%. The four-profile solution also showed clearer classification probabilities, with a minimum average posterior probability of 0.942, compared with 0.683 in the five-profile solution. These results indicate that the five-profile solution had greater classification overlap and included a relatively small additional class.

**Figure 2:**
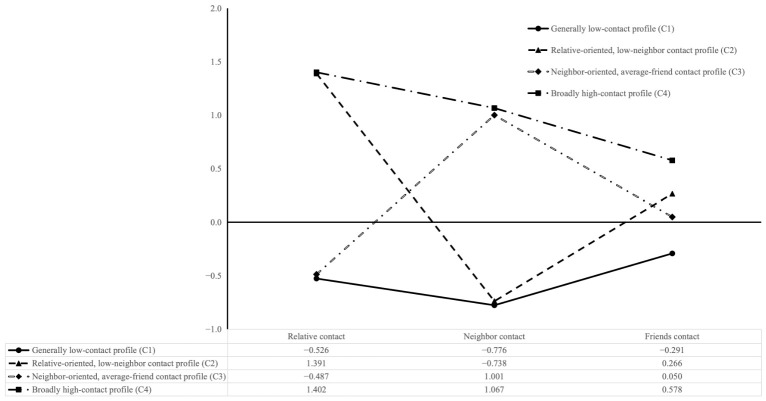
Estimated conditional means (standardized) of the four latent profiles of informal social contact.

For the four-profile model, both the LMR and BLRT were statistically significant, indicating that the four-profile solution improved model fit compared with the three-profile solution. Considering statistical fit, classification quality, class size, parsimony, and substantive interpretability, the four-profile solution was retained as the final model. The proportions of respondents in the four profiles are shown in Table 2, and the estimated conditional means of the four informal social contact profiles are presented in Figure 2.

Figure 2 shows that the conditional means of the four profiles on the three factors of informal social contact show similar trends for profiles 1 and 2 and profiles 3 and 4, and friend contact was less differentiated across profiles than relative and neighbor contact; in C3 it was close to the sample average. Profile 1 (C1) was named “Generally low-contact profile” based on its displayed characteristics and accounted for 44.1% of all respondents. Profile 2 (C2), which has a significantly higher conditional mean for relative contact than Profile 1 and is similar to Profile 4 and similar conditional means for neighbor contact and friend contact, named “Relative-oriented, low-neighbor contact profile”, accounted for 11.9% of all respondents. Profile 3 (C3) has a similar conditional mean of relative contact as profile 1, a similar conditional mean of neighbor contact as profile 4, and a similar conditional mean of friend contact as profiles 2 and 4, and is named “Neighbor-oriented, average-friend contact profile”, accounting for 28.2% of all respondents. Profile 4 (C4), which had higher mean values for all conditions than the other three profiles and accounted for 15.5% of all respondents, was named “Broadly high-contact profile” based on the characteristics of its scores.

### 3.2. Results of Multinomial Logistic Regression of Demographic Variables on 4 Latent Profiles

This study further examined whether exercise frequency and sociodemographic characteristics were associated with latent profile membership. The latent profile membership variable derived from the latent profile analysis was merged with the original dataset and treated as the dependent variable in a multinomial logistic regression model. The broadly high-contact profile, C4, was used as the reference category. Exercise frequency, gender, age, household registration status, educational level, income, and work characteristics were entered as predictors. For exercise frequency, “exercising every day” was used as the reference category. The odds ratios therefore indicate the likelihood of belonging to C1, C2, or C3 rather than C4, holding other variables constant. The results are presented in Table 3.

Compared with C4, household registration status was significantly associated with membership in C1, C2, and C3. The odds ratios were 0.56, 0.52, and 0.58, respectively, indicating that respondents with rural household registration were less likely to belong to these three profiles than to C4. In other words, rural respondents were more likely to be classified into the broadly high-contact profile, C4. Exercise frequency was also associated with profile membership. Compared with respondents who exercised every day, those who never exercised were more likely to belong to C1 rather than C4, OR = 2.70. This suggests that very low exercise participation was associated with the generally low-contact profile. Gender was significantly associated with membership in C1 and C3. Male respondents had higher odds of belonging to C1 and C3 rather than C4. Educational level was positively associated with membership in C1, indicating that respondents with higher educational attainment were more likely to belong to C1 than to C4. In addition, mentally demanding work was associated with higher odds of belonging to C2 rather than C4.

### 3.3. Comparison of Physical Exercise Frequency Scores Across Potential Profiles of Youth Informal Social Contact

The BCH method was used to compare exercise frequency scores across the four latent profiles. As shown in Table 4, the mean scores for exercise frequency in C1, C2, C3, and C4 were 3.07, 3.31, 3.00, and 3.20, respectively, corresponding approximately to exercising several times per month. Descriptively, C2 showed the highest mean exercise frequency, followed by C4, C1, and C3.

To control for the family-wise error rate, the Holm–Bonferroni correction was applied to the six pairwise BCH comparisons. After correction, only the difference between C2 and C3 remained statistically significant: χ^2^ = 7.503, raw *p* = 0.006, adjusted *p* = 0.036. Specifically, participants in C2 reported significantly higher exercise frequency than those in C3. The differences between C1 and C2 (χ^2^ = 5.129, raw *p* = 0.024, adjusted *p* = 0.120) and between C3 and C4 (χ^2^ = 3.906, raw *p* = 0.048, adjusted *p* = 0.192) were no longer statistically significant after correction. Other pairwise comparisons also remained nonsignificant. These results suggest that differences in exercise frequency across profiles were present descriptively but were limited after correction for multiple comparisons.

## 4. Discussion

### 4.1. Common Features and Heterogeneity in Informal Social Contact Profiles Among Young Adults

This study used latent profile analysis to examine three dimensions of informal social contact—contact with relatives, friends, and neighbors—among young adults in China. The results identified four profiles: C1, a generally low-contact profile; C2, a relative-oriented and low-neighbor-contact profile; C3, a neighbor-oriented profile with approximately average friend contact; and C4, a broadly high-contact profile. Among these profiles, C1 accounted for the largest proportion of the analytic sample, representing 44.1% of respondents.

C1, the generally low-contact profile, was more likely to include urban, highly educated, and male respondents. This pattern may reflect the combined influence of urban lifestyles, educational attainment, and gendered expectations. First, high population density, residential mobility, and the fast pace of urban life may contribute to relatively superficial and transient neighborhood relationships (Li et al., 2012; Zou et al., 2024), thereby reducing opportunities for contact with neighbors. Second, highly educated young adults often face demanding work or career-development pressures, which may limit the time and energy available to maintain regular contact with friends, relatives, and neighbors. Third, gender-role expectations may also matter. Men may be more likely to prioritize occupational competition and work-related responsibilities, potentially reducing their engagement in routine social and recreational interactions (Courtenay, 2000). Taken together, these factors may help explain why C1 was characterized by lower levels of contact across all three informal relationship domains.

C2, the relative-oriented and low-neighbor contact profile, was associated with mentally demanding work. This profile was characterized by relatively higher contact with relatives, lower contact with neighbors, and moderately above-average contact with friends. The relatively low level of neighbor contact may again be related to the weaker neighborhood ties commonly observed in urban settings. Meanwhile, young adults engaged in mentally demanding work may maintain contact with close relatives and friends as part of their routine coping and social interaction patterns. However, because the CGSS items measure contact frequency rather than the specific emotional or instrumental content of support, this interpretation should be understood as a possible explanation rather than direct evidence of support-seeking behavior. In addition, although C2 showed a relatively high mean level of exercise frequency, its difference from C4 was not statistically significant, and conclusions about exercise differences involving this profile should be interpreted cautiously after multiple-comparison correction.

C3, the neighbor-oriented profile with approximately average friend contact, showed relatively high contact with neighbors, low contact with relatives, and friend contact close to the sample average. This pattern may be related to spatial proximity and daily activity contexts. In young adulthood, geographic separation from relatives may reduce the frequency of everyday contact with extended family members. At the same time, neighborhood-based activities, shared residential environments, and local recreational spaces may increase opportunities for interaction with neighbors. Young adults may also form activity-based ties through shared interests, such as sports, volunteering, or community activities (Villatte et al., 2022; Lo Coco et al., 2019). Thus, for this group, neighbor contact may partly compensate for reduced routine contact with relatives, although the present data cannot determine whether these contacts provided emotional, instrumental, informational, or appraisal support.

C4, the broadly high-contact profile, was more prominent among young adults with rural household registration. This pattern may reflect the structural and cultural characteristics of rural communities. Rural areas are often characterized by relatively stable interpersonal networks, lower residential mobility, and stronger community familiarity. Residents may have lived near one another for long periods, which can facilitate repeated contact and stronger local ties (Sharp & Smith, 2003; Huang et al., 2025). In addition, shared cultural backgrounds, kinship ties, and community norms may reinforce reciprocal obligations and neighborhood familiarity. Relatives may also live in closer geographic proximity in rural settings, making routine contact with relatives more feasible. These conditions may contribute to a broader informal contact network involving friends, relatives, and neighbors.

Across the four profiles, friend contact tended to be less differentiated than contact with relatives and neighbors, suggesting that contact with friends may be a relatively common feature of young adults’ informal social networks. This pattern is consistent with developmental perspectives suggesting that, during the transition from adolescence to adulthood, individuals gradually move away from reliance on parents and relatives and increasingly construct identity, belonging, and companionship through peer relationships. Prior studies have suggested that adult friendships often involve reciprocal exchanges of support and shared activities (Beck et al., 2017), and friend groups based on common interests may strengthen social belonging (Villatte et al., 2022; Lo Coco et al., 2019). Nevertheless, the present findings should be interpreted as evidence of contact patterns rather than direct evidence of perceived social support. The CGSS items used in this study capture the frequency of meeting or engaging in social recreational activities, not the quality or function of support exchanged during these interactions.

### 4.2. Informal Social Contact Profiles and Exercise Frequency

The BCH analysis showed that the mean exercise frequency scores across the four profiles ranged from 3.00 to 3.31, corresponding approximately to exercising several times per month. After applying Holm–Bonferroni correction for the six pairwise BCH comparisons, only the difference between C2 and C3 remained statistically significant. Specifically, young adults in the relative-oriented and low-neighbor-contact profile reported higher exercise frequency than those in the neighbor-oriented profile with approximately average friend contact. Other pairwise differences were no longer statistically significant after correction.

The multinomial logistic regression further showed that individuals who never participated in physical activity were more likely to belong to C1 than to C4. This finding suggests that young adults with generally low levels of informal social contact may also be more likely to report very low exercise participation. However, this association should not be interpreted as evidence that low social contact causes physical inactivity. Rather, low contact and low exercise frequency may be jointly shaped by broader factors such as work pressure, urban lifestyle, time constraints, and limited opportunities for shared activities.

For C1, the combination of relatively weak contact with friends, relatives, and neighbors may indicate fewer opportunities for companionship, encouragement, and shared exercise contexts. From a social identity perspective, group membership and shared behavioral norms may be relevant to physical activity participation (Stevens et al., 2017). When young adults have fewer routine contacts with friends or local communities, they may have fewer opportunities to encounter exercise-related norms, invitations, or co-participation. However, the present study cannot directly test this mechanism because the CGSS items do not measure exercise-specific encouragement or support.

Descriptively, C2 and C4 showed relatively higher mean exercise frequency than C1 and C3. However, after multiple-comparison correction, the evidence for between-profile differences was limited, and only the C2–C3 difference remained statistically robust. This suggests that the relationship between informal social contact profiles and exercise frequency may be modest. One possible explanation is that contact with friends and relatives may provide more opportunities for planned or companion-based activities, whereas neighbor contact alone may not necessarily translate into exercise-related participation. Another possibility is that mentally demanding work among C2 respondents may increase the perceived value of exercise as a way to relax, manage stress, or maintain productivity (Kashihara et al., 2009). These explanations remain tentative and should be examined in future studies using measures of exercise-specific social support and longitudinal data.

The role of contact with relatives also requires cautious interpretation. Relative contact showed less variation across some profiles and appeared to be less clearly linked to exercise frequency than friend or neighbor contact. In contemporary China, urbanization and population mobility may increase geographic separation between young adults and extended family members (He, 2023; Wang, 2021). Such separation may reduce opportunities for face-to-face contact and co-participation in daily activities, including exercise. Moreover, family ties may express support through moral encouragement, financial assistance, or symbolic obligations rather than through direct participation in lifestyle behaviors. Therefore, the limited association between relative contact and exercise frequency may reflect the fact that relatives are less often involved in young adults’ daily exercise contexts.

In summary, the findings suggest that informal social contact profiles are associated with exercise frequency among young adults, but the magnitude and robustness of these differences are limited. Certain configurations of informal social contact, particularly those involving more frequent contact with friends and relatives, are modestly associated with higher exercise frequency. These findings may inform future research and interventions, but intervention implications should be framed cautiously and tested with more direct measures of exercise-specific support.

## 5. Conclusions and Limitations

### 5.1. Conclusions

This study identified four profiles of informal social contact among young adult respondents in the CGSS 2021 analytic sample: a generally low-contact profile, a relative-oriented and low-neighbor contact profile, a neighbor-oriented profile with approximately average friend contact, and a broadly high-contact profile. These profiles reflected heterogeneity in routine contact with relatives, friends, and neighbors within the analytic sample.

The findings also showed that profile membership was associated with exercise frequency. After correction for multiple BCH pairwise comparisons, only the difference between C2 and C3 remained statistically significant, indicating that respondents in the relative-oriented and low-neighbor contact profile reported higher exercise frequency than those in the neighbor-oriented profile with approximately average friend contact. In addition, multinomial logistic regression suggested that respondents who never exercised were more likely to belong to the generally low-contact profile than to the broadly high-contact profile.

Overall, the findings suggest an association between informal social contact profiles and exercise frequency within the analytic sample, but they do not support causal conclusions or nationally representative inferences. Friend contact appeared to be an important component of respondents’ informal contact networks, but it should not be described as a direct driver of exercise participation. Rather, the results suggest that exercise behavior in this sample may be embedded in broader patterns of everyday social contact. These findings may inform future research on peer-based and community-based exercise opportunities, but intervention implications should be tested using weighted, longitudinal, or experimental designs before being generalized to the broader population of young adults in China.

### 5.2. Limitations

Several limitations should be acknowledged. First, although the CGSS 2021 has national sample coverage, the present study used an unweighted analytic subsample of respondents aged 18–35 years with complete data on all study variables. Sampling weights and survey design features were not incorporated into the latent profile analysis, multinomial logistic regression, or BCH analyses. Therefore, the estimated profile proportions and profile-exercise associations should be interpreted as sample-specific findings rather than nationally representative estimates for all Chinese young adults. Future studies should examine the robustness of these findings using survey-weighted models, sensitivity analyses, or additional nationally representative datasets.

Second, all variables were measured using self-reported questionnaire items. Exercise frequency was assessed using a single item, which may not fully capture the intensity, duration, type, or context of physical activity. Similarly, the informal social contact indicators measured the frequency of contact or social recreational activities with relatives, friends, and neighbors, but did not directly assess the quality, emotional closeness, instrumental support, or informational support embedded in these relationships. Future research should incorporate more detailed measures of exercise behavior and social contact quality, including different exercise modalities and more specific dimensions of informal social relationships.

Third, the cross-sectional design of the CGSS 2021 limits causal inference. The observed associations cannot determine whether informal social contact influences exercise frequency, whether exercise participation increases social contact, or whether both are shaped by other social, demographic, or contextual factors. Future studies using longitudinal data, such as subsequent waves of the CGSS when available, could help assess whether informal social contact patterns and exercise behavior remain stable over time and clarify the directionality of their association.

## Figures and Tables

**Table 1:** Variable measurements and data processing.

Variable		Item Number	Measurement Questions	Data Processing
Latent profile indicators	Informal social contact	A30_6	In the past year, did you often get together in your free time with relatives who did not live with you?	Reverse scoring processing, standardization
		A31a	How often do you engage in social recreational activities with your neighbors?	Reverse scoring processing, standardization
		A31b	How often do you engage in social recreational activities with other friends?	Reverse scoring processed and merged, standardized
		A30_7	In the past year, did you often meet up with friends in your free time	
Exercise-related variable	Physical exercise	A30_9	In the past year, did you regularly participate in physical exercise in your free time	Reverse scoring processing, standardization
Demographic variables	Gender	A2	Gender	Re-valued, male = 1, female = 0
	Household registration status	A18_1	Your current residential properties registration status is Rural	Raw data with value 1
		A18_2	Your current residential properties registration status is non-rural	Combined as a residential property, taking the value 0
		A18_3	Your current residential properties registration status is Resident residential properties (formerly Rural Resident residential properties)	
		A18_4	Your current residential properties registration status is Resident residential properties (formerly Non-Rural residential properties)	
		A18_5	Military status	Number of cases too small, defined as missing values
		A18_6	Other	
		A18_7	No residential properties registration	
	Educational level	A7a_1	No education at all	Raw data with value 1
		A7a_2	Private schools, literacy classes	Raw data with value 2
		A7a_3	primary school	Raw data with value 3
		A7a_4	junior high school	Raw data with value 4
		A7a_5	secondary vocational technical school	Combined with a high school diploma, taking the value of 5
		A7a_6	general high school	
		A7a_7	technical secondary school	
		A7a_8	technical school	
		A7a_9	University College (Adult Higher Education)	Combined with a Bachelor’s Degree, taking the value of 6
		A7a_10	University College (Regular Higher Education)	
		A7a_11	University Bachelor’s Degree (Adult Higher Education)	
		A7a_12	University Bachelor’s Degree (Regular Higher Education)	
		A7a_13	Postgraduate and above	Raw data with value 7
		A7a_14	Other	This option does not exist in the sample
	Income	A8a	What was your personal gross income for the whole of last year (2020)	Raw data, natural logarithm conversion
	Type of work	L11_a	How often does heavy labor occur in your work?	Raw data, reverse scoring
		L11_b	How often does heavy mental labor occur in your work?	Raw data, reverse scoring

**Table 2:** Summary of fitting information for latent profile analysis.

Profile	*AIC*	*BIC*	*ABIC*	*pLMR*	*pBLRT*	*Entropy*	Group Size for Each Profile				
							1	2	3	4	5
2-Profile	15,064.5	15,119.7	15,087.9	<0.001	<0.001	0.897	1045 (56.4%)	808 (43.6%)			
3-Profile	14,975.6	15,052.9	15,008.4	<0.001	<0.001	0.890	998 (53.9%)	804 (43.4)	51 (2.8%)		
4-Profile	14,623.7	14,723.1	14,665.9	<0.001	<0.001	0.917	818 (44.1%)	221 (11.9%)	523 (28.2%)	291 (15.5%)	
5-Profile	9816.1	9937.6	9867.7	0.25	<0.001	0.834	146 (7.9%)	844 (45.5%)	351 (18.9%)	400 (21.6%)	112 (6.0%)

**Table 3:** Results of multinomial logistic regression of different demographic variables on four latent profiles and effect size.

Variable		C1			C2			C3		
		*β*	*OR*	*CI* (95%)	*β*	*OR*	*CI* (95%)	*β*	*OR*	*CI* (95%)
Factor										
Physical exercise participation frequency	Never	1.00	2.70 *	1.06–6.86	0.06	1.06	0.34–3.28	0.13	1.14	0.41–3.13
	Several times a year or less	0.57	1.78	0.75–4.22	−0.59	0.55	0.19–1.61	0.20	1.22	0.48–3.09
	Several times a month	0.15	1.16	0.52–2.59	−0.29	0.75	0.30–1.85	0.11	1.12	0.48–2.60
	Several times a week	0.18	1.20	0.54–2.69	−0.74	0.48	0.19–1.22	−0.10	0.90	0.38–2.15
	Every day	·	·	·	·	·	·	·	·	·
Covariate										
Gender		0.74	2.10 *	1.29–3.44	0.51	1.66	0.91–3.03	0.57	1.76 *	1.03–3.00
Age		−0.05	0.95	0.90–1.01	−0.05	0.95	0.89–1.02	−0.03	0.97	0.92–1.04
Residential properties		−0.58	0.56 *	0.34–0.92	−0.65	0.52 *	0.28–0.96	−0.55	0.58 *	0.34–0.98
Income		0.01	1.01	0.90–1.13	−0.03	0.97	0.84–1.12	0.03	1.03	0.92–1.04
Educational level		0.39	1.47 **	1.12–1.94	0.30	1.34	0.94–1.93	0.10	1.10	0.83–1.46
Type of work	Heavy labor	−0.12	0.89	0.66–1.20	−0.02	0.98	0.66–1.44	0.13	1.14	0.84–1.55
	Heavy mental work	0.14	1.15	0.90–1.48	0.43	1.54 *	1.13–2.08	0.05	1.05	0.80–1.38

Note. C4, the broadly high-contact profile, was used as the reference category. C1 = generally low-contact profile; C2 = relative-oriented, low-neighbor contact profile; C3 = neighbor-oriented, average-friend contact profile; * *p* < 0.05, ** *p* < 0.01.

**Table 4:** Comparison of differences in frequency of physical exercise participation across latent profiles.

Profiles	*M*	*SE*	Items	χ^2^	*Raw* *p*	*Holm–Bonferroni Adjusted p*	*Result*
Generally low-contact profile (C1)	3.07	0.05	C1 vs. C2	5.129	0.024	0.120	Nonsignificant
Relative-oriented, low-neighbor contact profile (C2)	3.31	0.09	C1 vs. C3	0.712	0.399	0.796	Nonsignificant
Neighbor-oriented, average-friend contact profile (C3)	3.00	0.06	C1 vs. C4	2.099	0.147	0.441	Nonsignificant
Broadly high-contact profile (C4)	3.20	0.08	C2 vs. C3	7.503	0.006	0.036	Significant
			C2 vs. C4	0.715	0.398	0.796	Nonsignificant
			C3 vs. C4	3.906	0.048	0.192	Nonsignificant

## Data Availability

The datasets generated during and/or analyzed during the current study are available in the Chinese National Survey Data Archive repository, URL: http://www.cnsda.org/index.php?r=projects/view&id=65635422 (accessed on 9 July 2025).
